# Use of sodium-glucose cotransporter-2 inhibitors and the risk for sudden cardiac arrest and for all-cause death in patients with type 2 diabetes mellitus

**DOI:** 10.1093/ehjcvp/pvac043

**Published:** 2022-07-27

**Authors:** Talip E Eroglu, Ruben Coronel, Coert J Zuurbier, Marieke Blom, Anthonius de Boer, Patrick C Souverein

**Affiliations:** Division of Pharmacoepidemiology and Clinical Pharmacology, Utrecht Institute for Pharmaceutical Sciences, Utrecht University, 3584 CS Utrecht, The Netherlands; Department of Cardiology, Copenhagen University Hospital—Herlev and Gentofte, 2900 Copenhagen, Denmark; Department of Experimental and Clinical Cardiology, Amsterdam UMC, Academic Medical Center, University of Amsterdam, Heart Centre, Amsterdam Cardiovascular Sciences, Meibergdreef 9, 1105 AZ Amsterdam, The Netherlands; Department of Anaesthesiology, Laboratory of Experimental Intensive Care and Anaesthesiology (L.E.I.C.A.), Amsterdam UMC, Location Academic Medical Centre (AMC), University of Amsterdam, Cardiovascular Sciences, 1105 AZ Amsterdam, The Netherlands; General Practice, Amsterdam UMC Location Vrije Universiteit Amsterdam, Boelelaan 1117, 1081 HV Amsterdam, The Netherlands; Health Behaviors & Chronic Diseases, Amsterdam Public Health Research Institute, 1105 BP Amsterdam, The Netherlands; Division of Pharmacoepidemiology and Clinical Pharmacology, Utrecht Institute for Pharmaceutical Sciences, Utrecht University, 3584 CS Utrecht, The Netherlands; Division of Pharmacoepidemiology and Clinical Pharmacology, Utrecht Institute for Pharmaceutical Sciences, Utrecht University, 3584 CS Utrecht, The Netherlands

**Keywords:** Sodium-glucose cotransporter-2 inhibitors, Pharmacoepidemiology, Sudden cardiac arrest

## Abstract

**Aims:**

Sodium-glucose cotransporter-2 inhibitors (SGLT-2is) are antidiabetic agents that can have direct cardiac effects by impacting on cardiac ion transport mechanisms that control cardiac electrophysiology. We studied the association between SGLT-2i use and all-cause mortality and the risk of sudden cardiac arrest (SCA) in patients with type 2 diabetes.

**Methods:**

Using data from the UK Clinical Practice Research Datalink, a cohort study among patients initiating a new antidiabetic drug class on or after January 2013 through September 2020 was conducted. A Cox regression with time-dependent covariates was performed to estimate the hazard ratios (HRs) of SCA and all-cause mortality comparing SGLT-2is with other second- to third-line antidiabetic drugs. Stratified analyses were performed according to sex, diabetes duration (<5 or ≥5 years), and the presence of cardiovascular disease.

**Results:**

A total of 152 591 patients were included. Use of SGLT-2i was associated with a reduced HR of SCA when compared with other second- to third-line antidiabetic drugs after adjustment for common SCA risk factors, although this association marginally failed to reach statistical significance [HR: 0.62, 95% confidence interval (95% CI): 0.38–1.01]. The HR of all-cause mortality associated with SGLT-2i use when compared with other second- to third-line antidiabetics was 0.43 (95% CI: 0.39–0.48) and did not vary by sex, diabetes duration, or the presence of cardiovascular disease. SGLT-2i use remained associated with lower all-cause mortality in patients without concomitant insulin use (HR: 0.56, 95% CI: 0.50–0.63).

**Conclusion:**

SGLT-2i use was associated with reduced all-cause mortality in patients with type 2 diabetes. The association between use of SGLT-2i and reduced risk of SCA was not statistically significant.

## Introduction

Sudden cardiac arrest (SCA) is a leading cause of mortality in Western societies, accounting for up to 50% of all cardiovascular deaths and up to 20% of all natural deaths.^1^ SCA is predominantly caused by cardiac arrhythmias in the setting of coronary artery disease, cardiomyopathy, or heart failure.^2^ The incidence rate of SCA has been reported to be three- to eight-fold higher in patients with diabetes and appears to be independent of cardiovascular risk factors.^3–5^ Consequently, there is a strong interest in minimizing SCA risk in patients with diabetes.

Sodium-glucose cotransporter-2 inhibitors (SGLT-2is) constitute a class of antidiabetic agents, used to reduce blood glucose by decreasing glucose reabsorption via inhibition of glucose cotransporter 2 in the kidney in type 2 diabetes patients.^6^ In addition to its role in glucose regulation, it is becoming increasingly clear that SGLT-2i has important off-target effects in the myocardium.^7–13^ Favourable effects on cardiac ion transport mechanisms that control cardiac electrophysiology (e.g. myocardial sodium hydrogen (Na^+^/H^+^) exchanger and the cardiac late sodium channel current) and the autonomic nervous system have been demonstrated.^7–13^ These mechanisms are particularly important as disturbances in these pathways may result in cardiac arrhythmias and SCA.^14–16^ To our knowledge, data on the risk of SCA upon use of SGTL-2i in a large unselected cohort of individuals with type 2 diabetes are sparse. Filling this knowledge gap is clearly needed given the rising prevalence of diabetes, the increased SCA risk,^3–5^ and the low post-arrest survival chances associated with diabetes.^17^ In this population-based cohort study, we investigated whether the use of SGLT-2i was associated with a reduced risk of SCA and all-cause mortality in patients with type 2 diabetes when compared with the use of other second- to third-line antidiabetic drugs. We used other second- to third-line antidiabetic drugs as the reference group since SGLT-2is are second- to third-line antidiabetic drugs.

## Methods

### Data sources

This study used data from the Clinical Practice Research Datalink (CPRD) GOLD, a primary care database shown to be representative of the general population in the UK.^18^ This database includes anthropometric data (e.g. body mass index) and lifestyle variables (e.g. smoking), as well as medical information (e.g. diagnoses and procedures, which are coded using the Read code classification^19^) and prescriptions written by general practitioners. The data collected in the CPRD have been shown to be of high quality and validity, since practices contribute to the CPRD only when their data quality is up to research standards.^18^ The register has been described in detail previously.^18^ The study protocol was approved by the Independent Scientific Advisory Committee of the CPRD (protocol number 20_000197). Patient informed consent was not necessary since the data were anonymized for research purposes.

### Study cohort

We defined a cohort of patients who initiated a new antidiabetic drug class on or after 1 January 2013 (the year in which the first SGLT-2i entered the market in UK) and those that switched or added an antidiabetic drug from a class that was not used before. The date of this new prescription was considered as the cohort entry date. The study period was between 2013 and 2020. These patients were followed until SCA, death, transfer out of the general practice or date of last data collection for the practice, whatever came first. We excluded patients initially treated with insulin because such patients are likely to have type 1 diabetes or advanced type 2 diabetes, and women with polycystic ovarian syndrome and gestational diabetes at baseline, as these are other indications for metformin therapy. Further, we excluded patients diagnosed with SCA at any time prior to cohort entry. All individuals were required to have at least 6 months’ valid history in the CPRD before the cohort entry date.

### Exposure

A time-varying exposure definition was used in which each person-day at any time during the follow-up period was classified into one of the following mutually exclusive categories: (1) SGLT-2i users (e.g. alone or in combination with other antidiabetic drugs), (2) users of other second- to third-line antidiabetics (defined as initiation of treatment with either thiazolidinediones, DPP-4 inhibitors, alpha-glucosidase inhibitors, GLP-1 analogues, prandial glucose regulators, combination of antidiabetic drugs, or switch to or add-on of an antidiabetic drugs, including insulin), and (3) users of metformin or sulfonylureas in monotherapy as these are prescribed as first-line drugs.

In order to do so, we first created treatment episodes for all individual classes of antidiabetic drugs. For each prescription, the quantity prescribed was divided by the daily dosage instruction in order to estimate the duration of each prescription. In the case of missing information, the median time between prescriptions was used to impute missing durations at patient-level. In the case of too few observations, the most frequent duration was used for the relevant product. Treatment episodes were constructed using the method of Gardarsdottir *et al.* (2010).^20^ A treatment episode was defined as a series of successive prescriptions, taking dose changes into account. If a new prescription for the same class of antidiabetic drug was issued before the theoretical end date of the previous prescription, then the number of overlapping days (units at home) was added to the end date of the subsequent prescription. If a different strength was prescribed, then the remaining days were reset to zero. We used a 30-day permissible gap between the end date of one prescription and the start of the next prescription, to allow for irregular use. If the next prescription started >30 days after the end of the old prescription, then we considered it a new treatment episode. We created separate treatment episodes for individual classes of antidiabetic drugs initially, and combined these episodes to allow for concurrent use of multiple classes of antidiabetics later.

### Outcome

Our primary outcome included SCA, which was defined using Read codes for the following conditions: cardiac arrest, sudden death, performed resuscitations, asystole, and cardio-respiratory arrest (see Supplementary material online, *Table S1* for Read codes used). Our secondary outcome was all-cause mortality, identified through the CPRD death date, which is shown to be registered accurately.^21^

### Covariates

We defined the following SCA risk factors as potential confounders as time-dependent variables at the start of each time interval: age, ischemic heart disease including myocardial infarction, heart failure, atrial fibrillation, peripheral artery disease, and the presence of an implantable cardioverter defibrillator (ICD) or pacemaker. Further, we assessed smoking status (current, recent, and past), body mass index (<30, ≥30 kg/m^2^) and haemoglobin A_1c_ (HbA_1c_) level (≤7.0, 7.0–8.0, >8) by using the most recent values at the start of each time interval. Finally, prescription of cardiovascular drugs was identified as a time-dependent variable 6 months prior to the start of each time interval, and was used to calculate the number of cardiovascular drugs used (0, 1, 2, ≥ 3).

### Statistical analysis

Cox regression analysis with time-dependent exposure and time-dependent covariates was conducted to estimate the hazard ratios (HRs) of SCA or all-cause mortality comparing SGLT-2is with other second- to third-line antidiabetics using three models: (1) adjusting for age and sex (model 1), (2) adjusting for all potential confounders (model 2), and (3) only adjusting for the covariates that were univariately associated with the outcome and that changed the beta-coefficient of the association between SGLT-2is and outcome by ≥5% (model 3). Next, we conducted two sensitivity analyses to assess the robustness of our findings. First, we repeated the primary analyses by taking a reference category without users of only concomitant metformin and sulfonylurea drugs, as these patients may be considered as having less advanced diabetes. Second, we repeated the analysis by not allowing concomitant use of insulin (a marker of advanced diabetes) at cohort entry and during follow-up. Finally, we performed stratified analysis according to sex, diabetes duration (<5 or ≥5 years), the presence of cardiovascular disease and heart failure to investigate a potential effect modification. The presence of interaction on a multiplicative scale between SGLT-2is and sex, diabetes duration, the presence of cardiovascular disease, and heart failure was estimated by consecutively including the cross-product of the two factors as a variable in the model.

The study population at baseline was described using descriptive statistics with continuous variables summarized by mean and standard deviation (SD) and categorical variables by absolute numbers and percentages.

## Results

### Baseline characteristics

**Figure 1:**
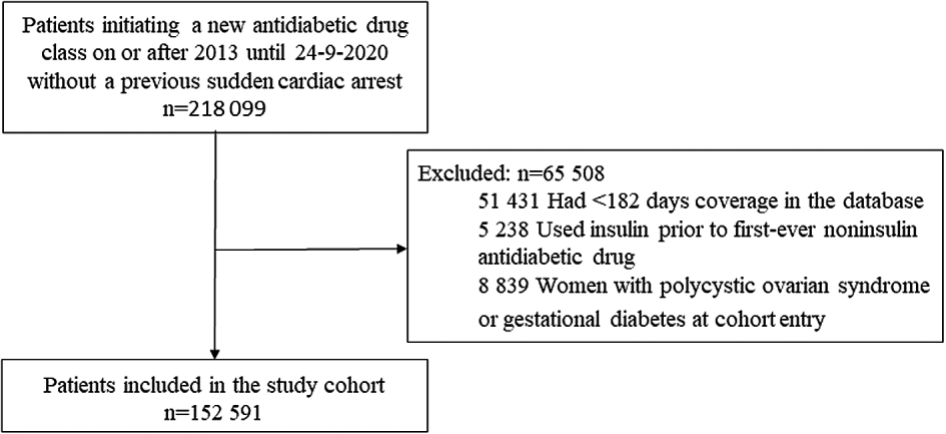
Flow chart depicting the inclusion of patients in the study cohort.

The cohort included 152 591 new users of antidiabetic drugs (*Figure** 1*). *Table** 1* presents the characteristics of the entire cohort stratified by antidiabetic drug use at cohort entry. Users of SGLT-2is were similar to users of other second- to third-line antidiabetic drugs with respect to sex, smoking status, and the number of cardiovascular drug prescriptions. However, users of SGLT-2is were younger, more likely to be obese, had higher HbA_1c_ levels, and a longer duration of diabetes compared with users of other second- to third-line antidiabetic users. Furthermore, the prevalence of cardiovascular comorbidities was lower, while the prevalence of insulin use was higher in users of SGLT-2is compared with users of other second- to third-line antidiabetic drugs.

**Table 1:** Baseline characteristics of users of sodium-glucose cotransporter-2 inhibitors, other second- to third-line drugs and first-line drugs

	Sodium-glucose cotransporter-2 inhibitors (*n* = 15 125)	Other second- to third-line drugs (*n* = 47 619)	First-line drugs (*n* = 89 847)	*P*-value
Age in years, mean (SD)	60.85 (10.02)	65.88 (12.76)	59.69 (14.79)	<0.001
Male, *n* (%)	9330 (61.7)	28 556 (60.0)	49 537 (55.1)	<0.001
Smoking status, *n* (%)				<0.001
Current	2652 (17.5)	8525 (17.9)	19 291 (21.5)	
Past	7653 (50.6)	23 667 (49.7)	37 886 (42.2)	
Never	4805 (31.8)	15 334 (32.2)	32 072 (35.7)	
Unknown	15 (0.1)	93 (0.2)	598 (0.7)	
BMI in kg/m^2^, *n* (%)				<0.001
<30	3940 (26.0)	21 020 (44.1)	32 090 (35.7)	
≥30	11 142 (73.7)	26 209 (55.0)	54 838 (61.0)	
Unknown	43 (0.3)	390 (0.8)	2919 (3.2)	
Haemoglobin A_1c_ level, *n* (%)				<0.001
≤ 7.0	649 (4.3)	4142 (8.7)	19 686 (21.9)	
7.1–8.0	2286 (15.1)	10 520 (22.1)	22 247 (24.8)	
>8.0	12 104 (80.0)	31 864 (66.9)	37 131 (41.3)	
Unknown	86 (0.6)	1093 (2.3)	10 783 (12.0)	
Duration of diabetes in years, mean (SD)	10.63 (5.04)	8.07 (5.36)	1.30 (2.74)	<0.001
Comorbidities, *n* (%)				
Ischemic heart disease including myocardial infarction	2388 (15.8)	8975 (18.8)	11 515 (12.8)	<0.001
Heart failure	500 (3.3)	2644 (5.6)	2872 (3.2)	<0.001
Atrial fibrillation	711 (4.7)	3984 (8.4)	5410 (6.0)	<0.001
Periphery arterial disease	420 (2.8)	1793 (3.8)	1864 (2.1)	<0.001
ICD/pacemaker	142 (0.9)	935 (2.0)	1011 (1.1)	<0.001
Cardiovascular drugs, *n* (%)				
Betablockers	3661 (24.2)	12 687 (26.6)	18 376 (20.5)	<0.001
Calcium channel blockers	4586 (30.3)	14 331 (30.1)	20 589 (22.9)	<0.001
Diuretics	3744 (24.8)	14 371 (30.2)	20 016 (22.3)	<0.001
Renin-angiotensin system inhibitors	10 292 (68.0)	29 021 (60.9)	34 223 (38.1)	<0.001
Statins	11 968 (79.1)	35 837 (75.3)	39 573 (44.0)	<0.001
Nitrates	1021 (6.8)	3827 (8.0)	5103 (5.7)	<0.001
Antiarrhythmic drugs	51 (0.3)	272 (0.6)	333 (0.4)	<0.001
Anticoagulants	745 (4.9)	3796 (8.0)	5430 (6.0)	<0.001
Number of concomitant cardiovascular drugs, *n* (%)				<0.001
0	1253 (8.3)	5101 (10.7)	31 715 (35.3)	
1	3043 (20.1)	9373 (19.7)	16 354 (18.2)	
2	4044 (26.7)	11 198 (23.5)	15 459 (17.2)	
≥3	6785 (44.9)	21 947 (46.1)	26 319 (29.3)	
Concomitant antidiabetic drug use				
Metformin	12 900 (85.3)	39 823 (83.6)	83 060 (92.4)	<0.001
Sulfonylureas	9321 (61.6)	31 386 (65.9)	6787 (7.6)	<0.001
Thiazolidinediones	1292 (8.5)	4579 (9.6)	0	<0.001
GLP-1 analogues	23 (0.2)	244 (0.5)	0	<0.001
DPP-4 inhibitors	3968 (26.2)	31 896 (67.0)	0	<0.001
SGLT-2 inhibitors	15 125 (100.0)	0	0	<0.001
Insulin	3415 (22.6)	3141 (6.6)	0	<0.001
Others	74 (0.5)	550 (1.2)	0	<0.001

### Association between SGLT-2i and sudden cardiac arrest

*Table* *2* shows the results related to SCA. The median (maximum) follow-up was 2.6 years (7.7), generating a total of 462 718.98 person-years. During follow-up, we identified 252 SCA events, corresponding to an overall incidence rate of 54.5 (95% CI: 48.1–61.6)/100 000 person-years. The incidence rate of SCA was lower in SGLT-2is users [3.3 (95% CI: 2.1–5.0)/10 000 person-years] compared with users of other second- to third-line antidiabetic drugs [7.1 (95% CI: 5.9–8.5)/10 000 person-years]. After adjusting for all the relevant confounders, use of SGLT-2i was associated with a lower hazard of SCA compared with users of other second- to third-line antidiabetic drugs (HR_model 2_: 0.63, 95% CI: 0.39–1.03; HR_model 3_: 0.62, 95% CI: 0.38–1.01), although this association just failed to reach statistical significance.

**Table 2:** Association between the use of sodium-glucose cotransporter-2 inhibitor and the hazard ratio of sudden cardiac arrest or all-cause mortality

Exposure	Events, no.	Person-years	Incidence rate per 10 000 person-years (95% CI)	Crude HR (95% CI)	Model 1 HR (95% CI)	Model 2 HR (95% CI)	Model 3* HR (95% CI)
Sudden cardiac arrest							
Second- to third-line antidiabetic drugs	116	163 975.43	7.1 (5.9–8.5)	1.0	1.0	1.0	1.0
Sodium-glucose cotransporter-2 inhibitors	20	61 405.982	3.3 (2.1–5.0)	0.46 (0.29–0.75)	0.60 (0.37–0.98)	0.63 (0.39–1.03)	0.62 (0.38–1.01)
Exposure	Events, no.	Person-years	Incidence rate per 1000 person-years (95% CI)	Crude HR (95% CI)	Model 1 HR (95% CI)	Model 2 HR (95% CI)	Model 3** HR (95% CI)
All-cause mortality							
Second- to third-line antidiabetic drugs	4816	164 094.05	29.3 (28.5–30.2)	1.0	1.0	1.0	1.0
Sodium-glucose cotransporter-2 inhibitors	431	61 421.325	7.0 (6.4–7.7)	0.24 (0.22–0.26)	0.42 (0.38–0.47)	0.44 (0.40–0.48)	0.43 (0.39–0.48)

CI, confidence interval; HR, hazard ratio.

Model 1: Adjusted for age and sex.

Model 2: Adjusted for age, sex, ischemic heart disease (including acute myocardial infarction), heart failure, atrial fibrillation, peripheral artery disease, duration of diabetes, smoking, HbA1C, BMI, ICD/pacemaker, and number of cardiovascular drugs.

Model 3*: Adjusted for variables that changed the association between SGLT-2 inhibitors and the outcome with ≥5% (age, ischemic heart disease including acute myocardial infarction, heart failure, atrial fibrillation, and duration of diabetes).

Model 3**: Adjusted for age, ischemic heart disease (including acute myocardial infarction), heart failure, atrial fibrillation, and duration of diabetes.

### Association between SGLT-2i and all-cause mortality

*Table* *2* shows the results related to all-cause mortality as our outcome. The incidence rate of all-cause mortality was lower in SGLT-2i users [7.0 (95% CI: 6.4–7.7)/1000 person-years] than in users of other second- to third-line antidiabetic drugs [29.3 (95% CI: 28.5–30.2)/1000 person-years]. After adjusting for all the relevant confounders, use of SGLT-2i was significantly associated with a reduced hazard of SCA compared with users of other second- to third-line antidiabetic drugs (HR_model 2_: 0.44, 95% CI: 0.40–0.48; HR_model 3_: 0.43, 95% CI: 0.39–0.48).

### Sensitivity analysis

The results of the sensitivity analyses are presented in Supplementary material online, *Tables S2* and *S3*. Overall, these sensitivity analyses yielded consistent findings, where the HRs did not vary when we repeated the analyses by taking a reference category without users of concomitant metformin and sulfonylurea drugs only (SCA: HR 0.60, 95% CI: 0.37–0.98; all-cause mortality: HR 0.42, 95% CI: 0.38–0.46, Supplementary material online, *Table S2*) or when we did not allow concomitant use of insulin (all-cause mortality: HR 0.56, 95% CI: 0.50–0.63, not reported for SCA due to too small numbers to obtain reliable results, Supplementary material online, *Table S3*).

### Stratified analysis

The results of the stratified analyses are summarized in Table 3 and presented in detail in Supplementary material online, *Tables S4–S8*. The HRs did not vary when stratifying on sex, duration of diabetes, or presence of cardiovascular disease, including heart failure. The HRs of SCA associated with SGLT-2i use in patients with type 2 diabetes with cardiovascular disease are presented in Supplementary material online, *Table S8*. After adjusting for common SCA risk factors, use of SGLT-2i was associated with a reduced hazard of SCA (HR: 0.60, 95% CI: 0.36–0.98). The results in patients with type 2 diabetes without cardiovascular disease are not reported due to small numbers. Also, other stratified analyses in relation to SCA as our outcome are not reported because of too small numbers to get meaningful results.

**Table 3:** Hazard ratio of all-cause mortality upon use of sodium-glucose cotransporter-2 inhibitors stratified according to sex, heart failure, cardiovascular disease, and diabetes duration

Stratification	Adjusted HR (95% CI)	*P*-value interaction
**Sex**		
Women	0.42 (0.36–0.50)	0.715
Men	0.45 (0.40–0.51)	
**Diabetes duration**		
<5 year	0.46 (0.36–0.60)	0.957
≥5 year	0.44 (0.40–0.49)	
**Cardiovasculair disease**		
Absent	0.36 (0.23–0.55)	0.161
Present	0.44 (0.39–0.48)	
**Heart failure**		
Absent	0.45 (0.40–0.50)	0.263
Present	0.41 (0.30–0.55)	

CI, confidence interval; HR, hazard ratio.

Hazard ratios were for age, sex, ischemic heart disease (including acute myocardial infarction), heart failure, atrial fibrillation, peripheral artery disease, duration of diabetes, smoking, HbA1C, body mass index, ICD/pacemaker, and number of cardiovascular drugs. When stratified according to sex, we did not adjust for sex. When stratified according to heart failure, we did not adjust for heart failure.

## Discussion

In this population-based cohort study, we found that (1) the use of SGLT-2i was associated with a reduced hazard of SCA compared with other second- to third-line antidiabetic drugs in patients with type 2 diabetes after adjustment for common SCA risk factors, although this association failed to reach statistical significance; and (2) the use of SGLT-2i was associated with a reduced hazard of all-cause mortality compared with other second- to third-line antidiabetic drugs in patients with type 2 diabetes. This reduced hazard of all-cause mortality occurred in both sexes and appears to be independent of diabetes duration and cardiovascular disease, including heart failure. Finally, use of SGLT-2i remained associated with lower all-cause mortality when we did not allow concomitant use of insulin.

Our finding that SGLT-2is were associated with reduced all-cause mortality is supported by previous studies.^22–25^ Chen *et al.* found using a longitudinal observational database that SGLT-2is were significantly associated with a reduced rate of all-cause mortality (HR: 0.55) and new-onset arrhythmias (HR: 0.83) compared with no use of SGLT-2is.^22^ However, data on important risk factors for SCA, such as myocardial infarction and heart failure, were not included in those analyses, and no direct adjustments for these factors were performed. Also, laboratory data such as HbA1_C_ and body mass index were not available. Fernandes *et al.* conducted a meta-analysis of randomized clinical trials (RCTs) and reported that SGLT-2is were associated with a reduced risk of sudden cardiac death (odds ratio: 0.72) in patients with type 2 diabetes.^23^ In a RCT, Curtain *et al.* demonstrated that the SGLT-2i dapagliflozin reduced the risk of ventricular arrhythmias, cardiac arrest, or sudden death in patients with heart failure and reduced ejection fraction^24^ in comparison to placebo. Also, other RCTs of SGLT-2is^23–26^ investigated the effect of SGLT-2is by comparing with placebo, and therefore did not provide head-to-head comparison with other antidiabetic drugs. In our study, we compared the effect with that of other antidiabetic drugs.

Previous studies demonstrated that diabetes is associated with increased SCA risk.^3–5^ Consequently, confounding by indication or disease duration (time lag bias)^27^ must be considered in our study. In our study, we therefore compared SGLT-2is (second- to third-line treatment strategy) with other second- to third-line antidiabetic drugs. However, although metformin is recommended as the preferred initial antidiabetic drug in patients with type 2 diabetes,^28–29^ guidelines from the European Society of Cardiology suggest initiation of SGLT-2is in patients with type 2 diabetes who are at high cardiovascular risk irrespective of prior metformin treatment.^30^ Confounding by disease duration may occur as a shorter duration of diabetes in users of SGLT-2is may be associated with lower SCA and all-cause incidence. In our study, however, the prevalence of metformin use at baseline was equal among patients that initiated SGLT-2is or other second- to third-line antidiabetic drugs (*Table** 1*), making this confounding unlikely. It is more likely that patients using SGLT-2is might have more severe diabetes than patients using other second- to third-line antidiabetic drugs in our study, since users of SGLT-2is had a longer duration of diabetes and a higher prevalence of insulin use compared with users of other second- to third-line antidiabetic drugs at cohort entry in our study (*Table** 1*). Nonetheless, despite being in a more advanced stage of diabetes than users of other second- to third-line antidiabetic drugs, users of SGLT-2is still had lower all-cause mortality and an almost significant lower SCA risk. Furthermore, our findings that SGLT-2is were associated with lower all-cause mortality, independent of diabetes duration and insulin use, provided additional support for the notion that our observed association was probably a drug effect. Although we did not find a statistically significant association between SGLT-2is and SCA in our study, a trend towards a reduced SCA rate was observed. It is likely that a lack of statistical power masked an otherwise significant association (i.e. in only 20 users of SGLT-2is SCA occurred during the study period), which is highlighted by the wide confidence interval.

Previous large-scale clinical trials have consistently demonstrated that SGLT-2is have their beneficial effects equally in patients with HF with and without type 2 diabetes,^31–34^ implying that these outcomes are independent of their anti-diabetic effects. Despite reductions in blood pressure and body weight following treatment with SGLT-2is,^35^ it is unlikely that the beneficial effects of SGLT-2is can be ascribed to a general reduction in traditional risk factors (e.g. glycaemic status, body weight, and blood pressure) since no beneficial effects of empagliflozin on the incidence of myocardial infarction and stroke are described.^25^ Consequently, it was hypothesized that the beneficial cardiovascular effects of SGLTis are, at least in part, caused by direct cardiac effects.^7, 36^ These direct cardiac effects are all related to the attenuation of disturbances in Ca^2+^ and Na^+^ ion homoeostasis by SGLTis.^36^ Indeed, empagliflozin directly inhibits the cardiac sodium-hydrogen exchanger (NHE), reduced cytosolic Na^+^ ([Na^+^]_c_) and Ca^2+^ ([Ca^2+^]_c_) concentrations, and increased mitochondrial Ca^2+^ concentration ([Ca^2+^]_m_) in cardiomyocytes.^7^ It was hypothesized that the observed decreased [Ca^2+^]_c_ and increased [Ca^2+^]_m_ are likely to have occurred secondary to the decrease in cytosolic Na^+^ via the sarcolemmal and mitochondrial Na^+^/Ca^2+^ exchanger (NCX), respectively.^36^ An increase in [Ca^2+^]_c_ may result in delayed afterdepolarizations leading to triggered activity, and/or impaired cell‐to‐cell transmission through gap-junctional uncoupling.^14^ These changes may culminate in arrhythmias and increase the risk for SCA.^14^ Because diabetes may also result in intracellular Na^+^ and Ca^2+^ loading,^37^ SGLT-2is could modulate SCA risk in these patients by reducing [Ca^2+^]_c._

In our study, we were not able to study each specific SGLT-2i drug separately due to small numbers. However, Uthman et al. have demonstrated earlier that the effects on [Na^+^]_c_ and [Ca^2+^]_c_ are a drug class specific effects.^38^ Therefore, it is likely that our finding regarding the reduced SCA rate is also a class effect. Besides the effects of SGLT-2is on NHE, SGLT-2is may also decrease [Ca^2+^]_c_ by reducing Ca^2+^/calmodulin-dependent kinase II (CaMKII) activity and CaMKII-dependent Ca^2+^ leak from the sarcoplasmic reticulum.^39^ Because CaMKII is elevated in diabetes,^40^ SGLT-2i use may directly reduce Ca^2+^ release and the risk of arrhythmias and SCA in patients with type 2 diabetes.^39^ Finally, attenuation of disturbances in ion homoeostasis can also be mediated through SGLT2i inhibition of the late component of the sodium current (late-I_Na_).^12,41^ Diabetes-related increases in reactive oxygen species and fatty acid metabolites may induce late-I_Na_ and action potential prolongation and increase the risk of Torsades de Pointes arrhythmia, ventricular fibrillation, and SCA.^15^ Thus, SGLT-2is may reduce SCA risk by inhibition of I_Na, L._ SGLT-2is may also have a favourable effect on the autonomic nervous system with a decrease in the cardiac sympathetic activity and an increase in the parasympathetic activity compared with placebo.^13^ Although further studies are needed to confirm these findings, this is an important mechanism since diabetes-induced cardiac autonomic neuropathy could lead to an imbalance of the sympathetic and parasympathetic control of the cardiac electrophysiology. Finally, indirect effects of SGLT2is may also decrease cardiac arrhythmia and SCA through attenuation of remodeling, fibrosis, and hypertrophy^8–11^ through improvements in ion homoeostasis.^36^

### Limitations

The Read codes used to identify SCAs in CPRD have not been validated previously, which may result in misclassification of the outcome by including SCAs from non-cardiac causes or that SCAs in the setting of acute myocardial infarction were classified as myocardial infarction events in the registries. However, the majority of the information recorded in CPRD generally shows to be of high quality and validity.^18^ Second, misclassification of the outcome could also occur if SCAs in the setting of acute myocardial infarction were classified as myocardial infarction events in the registries. We expect, however, that such possible misclassification is probably similarly distributed between the exposure groups. Third, prescriptions in the CPRD represent those issued by general practitioners, and thus misclassification of exposure may have occurred if patients were also treated by specialists. However, in the UK, the management of type 2 diabetes occurs almost entirely through primary care, and thus it is likely that such misclassification is minimal in our study.

Another limitation is that we had no data on important risk factors such as left ventricular ejection fraction and ventricular premature beats to include in our statistical model. However, considering the highly unpredictable way in which SCA occurs, it is difficult, if not impossible, to obtain such data shortly before SCA occurrence in a uniform manner across the studied population. Further, <1% of the patients had a record of with prior ventricular tachyarrhythmias. Consequently, we chose not to include this variable in our model as this would not change our conclusion due to the very low number of patients with registered prior ventricular tachyarrhythmias. A possible explanation for the low number of prior ventricular tachyarrhythmias in our data could be that this variable is too specific and therefore not registered in a primary care database.

Finally, possible confounding by indication must be considered since diabetes itself is a known risk factor for SCA and mortality.^3–5^ To minimize this potential bias, we used an active comparator design in which SGLT-2i was compared with other second- to third-line antidiabetic drugs. Further, our stratified analysis according diabetes duration and sensitivity analysis in which concomitant insulin use was not allowed provided additional support for the notion that our observed associations were due to SGLT-2i use. Moreover, our models were also adjusted for duration of diabetes, HbA_1c_, and body mass index.

## Supplementary Material

pvac043_Supplemental_File

## Data Availability

No data are available. The authors are contractually not allowed to share raw CPRD data in the public domain.

## Conclusion

Use of SGLT-2i is associated with a reduced risk of all-cause mortality in patients with type 2 diabetes. The association between use of SGLT-2i and a reduced risk of SCA was not statistically significant.
