# Supplementary material for: Use of sodium-glucose cotransporter-2 inhibitors and the risk for sudden cardiac arrest and for all-cause death in patients with type 2 diabetes mellitus
Source: Eur Heart J Cardiovasc Pharmacother. 2022 Jul 27;9(1):18–25. doi: 10.1093/ehjcvp/pvac043 (PMC9780744; doi:10.1093/ehjcvp/pvac043)
Supplement: pvac043_Supplemental_File [file pvac043_supplemental_file.docx]

| Supplementary Table 1: definition and codes used to define sudden cardiac arrest | |
| --- | --- |
| Sudden cardiac arrest | Read codes |
| cardiac arrest | medcode: 2099, 5925, 21195, 33899, 25583, 49882 |
| Sudden death | medcode: 23075, 73130, 6811, 23830 |
| Asystole | medcode: 33402 |
| Cardio-respiratory arrest | medcode: 25407 |
| All resuscitations | medcode: 28236, 39285, 23335, 51140, 96801, 99337 |

| Supplementary Table 2. Association between the use of sodium-glucose cotransporter-2 inhibitor and the hazard ratio of sudden cardiac arrest or all-cause mortality compared with second- to third-line antidiabetic drugs without users of concomitant metformin and sulfonylurea drugs alone | | | | | | |
| --- | --- | --- | --- | --- | --- | --- |
| Sudden cardiac arrest | | | | | | |
| Exposure | Events, No. | Person-years | Incidence rate per 10,000 person-years (95% CI) | Crude  HR (95% CI) | Model 1  HR (95% CI) | Model 2  HR (95% CI) |
| Second- to third-line antidiabetic drugs | 97 | 123116.56 | 7.88 (6.46-9.61) | 1.0 | 1.0 | 1.0 |
| Sodium-glucose cotransporter-2 inhibitors | 20 | 61405.982 | 3.26 (2.10-5.05) | 0.42 (0.26-0.67) | 0.55 (0.34-0.90) | 0.60 (0.37-0.98) |
| All-cause mortality | | | | | | |
|  | Events, No. | Person-years | Incidence rate per 1,000 person-years (95% CI) | Crude  HR (95% CI) | Model 1 HR (95% CI) | Model 2 HR (95% CI) |
| Second- to third-line antidiabetic drugs | 4057 | 123221.31 | 32.9 (31.9-34.0) | 1.0 | 1.0 | 1.0 |
| Sodium-glucose cotransporter-2 inhibitors | 431 | 61421.325 | 7.02 (6.38-7.71) | 0.21 (0.19-0.23) | 0.39 (0.36-0.44) | 0.42 (0.38-0.46) |
| CI, confidence interval; HR, hazard ratio  Model 1: Adjusted for age and sex  Model 2: Adjusted for age, sex, ischemic heart disease (including acute myocardial infarction), heart failure, atrial fibrillation, peripheral artery disease, duration of diabetes, smoking, HbA_1c_, body mass index, ICD/pacemaker and number of cardiovascular drugs | | | | | | |

| Supplementary Table 3. Association between the use of sodium-glucose cotransporter-2 inhibitor and the hazard ratio of all-cause mortality compared with second- to third-line antidiabetic drugs in absence of insulin | | | | | | |
| --- | --- | --- | --- | --- | --- | --- |
| Exposure | Events, No. | Person-years | Incidence rate per 1,000 person-years (95% CI) | Crude  HR (95% CI) | Model 1  HR (95% CI) | Model 2  HR (95% CI) |
| Second- to third-line antidiabetic drugs | 2873 | 132771.89 | 21.64 (20.86-22.44) | 1.0 | 1.0 | 1.0 |
| Sodium-glucose cotransporter-2 inhibitors | 321 | 50846.656 | 6.31 (5.66-7.04) | 0.29 (0.26-0.33) | 0.54 (0.48-0.60) | 0.56 (0.50-0.63) |
| CI, confidence interval; HR, hazard ratio  Model 1: Adjusted for age and sex  Model 2: Adjusted for age, sex, ischemic heart disease (including acute myocardial infarction), heart failure, atrial fibrillation, peripheral artery disease, duration of diabetes, smoking, HbA_1c_, body mass index, ICD/pacemaker and number of cardiovascular drugs | | | | | | |

| Supplementary Table 4. Association between the use of sodium-glucose cotransporter-2 inhibitor and the hazard ratio of all-cause mortality compared with second- to third-line antidiabetic drugs stratified by sex | | | | | | |
| --- | --- | --- | --- | --- | --- | --- |
| Exposure | Events, No. | Person-years | Incidence rate per 1,000 person-years (95% CI) | Crude  HR (95% CI) | Model 1  HR (95% CI) | Model 2  HR (95% CI) |
| Women | | | | | | |
| Second- to third-line antidiabetic drugs | 2006 | 66500.578 | 30.17 (28.87-31.51) | 1.0 | 1.0 | 1.0 |
| Sodium-glucose cotransporter-2 inhibitors | 142 | 22599.748 | 6.28 (5.33-7.41) | 0.21 (0.18-0.25) | 0.41 (0.34-0.49) | 0.42 (0.36-0.50) |
| Men | | | | | | |
| Exposure | Events, No. | Person-years | Incidence rate per 1,000 person-years (95% CI) | Crude  HR (95% CI) | Model 1  HR (95% CI) | Model 2  HR (95% CI) |
| Second- to third-line antidiabetic drugs | 2810 | 97593.476 | 28.79 (27.75-29.88) | 1.0 | 1.0 | 1.0 |
| Sodium-glucose cotransporter-2 inhibitors | 289 | 38821.577 | 7.44 (6.63-8.35) | 0.26 (0.23-0.29) | 0.43 (0.38-0.49) | 0.45 (0.40-0.51) |
| P-value interaction: 0.715  CI, confidence interval; HR, hazard ratio  Model 1: Adjusted for age  Model 2: Adjusted for age, ischemic heart disease (including acute myocardial infarction), heart failure, atrial fibrillation, peripheral artery disease, duration of diabetes, smoking, HbA_1c_, body mass index, ICD/pacemaker and number of cardiovascular drugs | | | | | | |

| Supplementary Table 5. Association between the use of sodium-glucose cotransporter-2 inhibitor and the hazard ratio of all-cause mortality compared with second- to third-line antidiabetic drugs stratified by diabetes duration | | | | | | |
| --- | --- | --- | --- | --- | --- | --- |
| Exposure | Events, No. | Person-years | Incidence rate per 1,000 person-years (95% CI) | Crude  HR (95% CI) | Model 1  HR (95% CI) | Model 2  HR (95% CI) |
| Diabetes duration (< 5 years) | | | | | | |
| Second- to third-line antidiabetic drugs | 1003 | 45687.25 | 21.95 (20.64-23.36) | 1.0 | 1.0 | 1.0 |
| Sodium-glucose cotransporter-2 inhibitors | 61 | 11805.9 | 5.17 (4.02-6.64) | 0.25 (0.19-0.32) | 0.44 (0.34-0.56) | 0.46 (0.36-0.60) |
| Diabetes duration (≥ 5 years) | | | | | | |
| Exposure | Events, No. | Person-years | Incidence rate per 1,000 person-years (95% CI) | Crude  HR (95% CI) | Model 1  HR (95% CI) | Model 2  HR (95% CI) |
| Second- to third-line antidiabetic drugs | 3813 | 118406.8 | 32.20 (31.20-33.24) | 1.0 | 1.0 | 1.0 |
| Sodium-glucose cotransporter-2 inhibitors | 370 | 49615.425 | 7.46 (6.73-8.26) | 0.23 (0.21-0.2~~6~~) | 0.43 (0.39-0.48) | 0.44 (0.40-0.49) |
| P-value interaction: 0.957 CI, confidence interval; HR, hazard ratio  Model 1: Adjusted for age and sex  Model 2: Adjusted for age, sex, ischemic heart disease (including acute myocardial infarction), heart failure, atrial fibrillation, peripheral artery disease, duration of diabetes, smoking, HbA_1c_, body mass index, ICD/pacemaker and number of cardiovascular drugs | | | | | | |

| Supplementary Table 6. Association between the use of sodium-glucose cotransporter-2 inhibitor and the hazard ratio of all-cause mortality compared with second- to third-line antidiabetic drugs stratified by presence of cardiovascular disease | | | | | | |
| --- | --- | --- | --- | --- | --- | --- |
| Absence of cardiovascular disease | | | | | | |
| Exposure | Events, No. | Person-years | Incidence rate per 1,000 person-years (95% CI) | Crude  HR (95% CI) | Model 1  HR (95% CI) | Model 2  HR (95% CI)* |
| Second- to third-line antidiabetic drugs | 308 | 14185.788 | 21.71 (19.42-24.28) | 1.0 | 1.0 | 1.0 |
| Sodium-glucose cotransporter-2 inhibitors | 22 | 4613.3224 | 4.77 (3.14-7.24) | 0.22 (0.14-0.34) | 0.34 (0.22-0.53) | 0.36 (0.23-0.55) |
| Presence of cardiovascular disease | | | | | | |
| Exposure | Events, No. | Person-years | Incidence rate per 1,000 person-years (95% CI) | Crude  HR (95% CI) | Model 1  HR (95% CI) | Model 2  HR (95% CI)** |
| Second- to third-line antidiabetic drugs | 4508 | 149908.27 | 30.07 (29.21-30.96) | 1.0 | 1.0 | 1.0 |
| Sodium-glucose cotransporter-2 inhibitors | 409 | 56808.003 | 7.20 (6.53-7.93) | 0.24 (0.22-0.26) | 0.43 (0.39-0.47) | 0.44 (0.39-0.48) |
| P-value interaction: 0.161  CI, confidence interval; HR, hazard ratio  Model 1: Adjusted for age and sex  Model 2*: Adjusted for age, sex, duration of diabetes, smoking, HbA_1c_, body mass index  Model 2**: Adjusted for age, sex, ischemic heart disease (including acute myocardial infarction), heart failure, atrial fibrillation, peripheral artery disease, duration of diabetes, smoking, HbA_1c_, body mass index, ICD/pacemaker and number of cardiovascular drugs | | | | | | |

| Supplementary Table 7. Association between the use of sodium-glucose cotransporter-2 inhibitor and the hazard ratio of all-cause mortality compared with second- to third-line antidiabetic drugs stratified by presence of heart failure | | | | | | |
| --- | --- | --- | --- | --- | --- | --- |
| Absence of heart failure | | | | | | |
| Exposure | Events, No. | Person-years | Incidence rate per 1,000 person-years (95% CI) | Crude  HR (95% CI) | Model 1  HR (95% CI) | Model 2  HR (95% CI) |
| Second- to third-line antidiabetic drugs | 3827 | 154437.67 | 24.78 (24.01-25.58) | 1.0 | 1.0 | 1.0 |
| Sodium-glucose cotransporter-2 inhibitors | 386 | 59718.527 | 6.46 (5.85-7.14) | 0.26 (0.23-0.29) | 0.44 (0.40-0.49) | 0.45 (0.40-0.50) |
| Presence of heart failure | | | | | | |
| Exposure | Events, No. | Person-years | Incidence rate per 1,000 person-years (95% CI) | Crude  HR (95% CI) | Model 1  HR (95% CI) | Model 2  HR (95% CI) |
| Second- to third-line antidiabetic drugs | 989 | 9656.386 | 102.42 (96.23-109.01) | 1.0 | 1.0 | 1.0 |
| Sodium-glucose cotransporter-2 inhibitors | 45 | 1702.7981 | 26.43 (19.73-35.39) | 0.26 (0.19-0.35) | 0.42 (0.31-0.57) | 0.41 (0.30-0.55) |
| P-value interaction: 0.263  CI, confidence interval; HR, hazard ratio  Model 1: Adjusted for age and sex  Model 2: Adjusted for age, sex, ischemic heart disease (including acute myocardial infarction), atrial fibrillation, peripheral artery disease, duration of diabetes, smoking, HbA_1c_, body mass index, ICD/pacemaker and number of cardiovascular drugs | | | | | | |

| Supplemental Table 8. Association between the use of sodium-glucose cotransporter-2 inhibitor and the hazard ratio of sudden cardiac arrest in patient with type 2 diabetes with cardiovascular disease | | | | | | |
| --- | --- | --- | --- | --- | --- | --- |
| Sudden cardiac arrest | | | | | | |
| Exposure | Events, No. | Person-years | Incidence rate per 10,000 person-years (95% CI) | Crude  HR (95% CI) | Model 1  HR (95% CI) | Model 2  HR (95% CI) |
| Second- to third-line antidiabetic drugs | 113 | 149793.48 | 7.54 (6.27-9.07) | 1.0 | 1.0 | 1.0 |
| Sodium-glucose cotransporter-2 inhibitors | 19 | 56792.70 | 3.35 (2.13-5.25) | 0.45 (0.27-0.73) | 0.57 (0.35-0.93) | 0.60 (0.36-0.98) |
| CI, confidence interval; HR, hazard ratio  Model 1: Adjusted for age and sex  Model 2: Adjusted for age, sex, ischemic heart disease (including acute myocardial infarction), heart failure, atrial fibrillation, peripheral artery disease, duration of diabetes, smoking, HbA_1c_, body mass index, ICD/pacemaker and number of cardiovascular drugs | | | | | | |
